# How point-of-care Ultrasound became an essential part of the assessment in the Emergency Department

**DOI:** 10.1016/j.radcr.2022.03.092

**Published:** 2022-05-09

**Authors:** Mahmoud El-Hussein, Cyma Hamieh, Maxime Gautier

**Affiliations:** a Emergency Medicine Department, APHP, Lariboisière Hospital, Paris, France; b Family Medicine Department, Lebanese American University Medical Center, Beirut, Lebanon

**Keywords:** ultrasound, emergency ultrasound, POCUS, FAST, Aortic Dissection, Emergency Medicine

## Abstract

From its first use in medicine, ultrasonography has been an excellent non-invasive diagnostic tool. The use of ultrasound increased dramatically especially in the last decade, as it is a painless, safe, and widely accessible, especially with the development of pocket ultrasound machines. In addition, it is crucial in promptly diagnosing unstable patients in emergency settings. Currently, emergency physicians are leaning more towards ultrasound, fellowships and university courses are created around the globe to teach this essential skill. This article highlights the importance of ultrasound in the hands of emergency physicians while presenting a life-threatening diagnosis that could have been easily missed if it wasn't for the use of ultrasound. Cases like these are frequently seen in the emergency departments, and it is when blinded by a tunnel vision, and anchoring bias, that these serious diagnoses can be left undiagnosed. Many individuals in the medical community are against the use of ultrasound by untrained physicians, but it remains highly recommended that emergency physicians get a proper training on the use of this tool for it is of great value in the emergency department.

## Introduction

From its first introduction, ultrasonography has been an excellent non-invasive diagnostic tool. In the late 1990s, ultrasound was put in use in emergency settings with the emergence of Point-Of-Care-Ultrasonography (POCUS). Its use increased dramatically and gained popularity as it is safe, painless and easily accessible. POCUS is a quick technique, that has proven its efficacy in assessing patients in emergency settings, and from which emerged Focused Cardiac Ultrasound (FoCUS) that quickly identifies cardiac emergencies such as pericardial effusions or tamponade, and the Focused Assessment with Sonography for Trauma (FAST), to search for abdominal fluid post traumatic injuries [1]. In POCUS, physicians search for the presence of critical findings in vital body organs such as the heart, lungs, and vessels... Myocardial infarctions, tamponade, pericardial effusion, pneumothorax, aortic dissections, and many other diagnoses can be detected by well-trained emergency physicians [1]. As its importance was worldwide recognized, not only residency programs integrated ultrasound in their curriculum, but also medical schools during medical student's clerkships [3]. In 2001, POCUS training and credentialing for emergency physicians were first published by the American College of Emergency Physicians (ACEP), and were updated regularly to adapt for the rapid increase in ultrasound in the emergency departments [4]. In addition, Ultrasound Fellowship training programs were created for emergency physicians interested in expanding their knowledge in ultrasound [5]. Also, Ultrasound workshops and teaching are always integrated in almost every emergency medicine conference around the world [6].

This article highlights the importance of ultrasound while giving an example of an aortic dissection that would have been missed if it wasn't for the use of ultrasound in the emergency department.

## Case presentation

Abdominal pain is a common chief complaint that is seen frequently in the emergency department. Although it can refer to other organs such as the heart, lungs or the kidney, abdominal pain can be attributable to much simpler and common diagnoses such as simple gastritis or irritable bowel syndrome especially in the younger population, when EKG and all the blood tests including troponin are normal.

A 34-year-old patient, with a history of hypertension on dual anti-hypertensive therapy, well followed by nephrology, presented to the emergency department for acute onset epigastric pain. The patient described the pain as continuous, burning, non-radiating of moderate intensity. Besides a high blood pressure of 200/84 mmHg, all other vital signs were normal. The patient had an epigastric tenderness with no other pertinent physical finding.

**Fig. 1:**
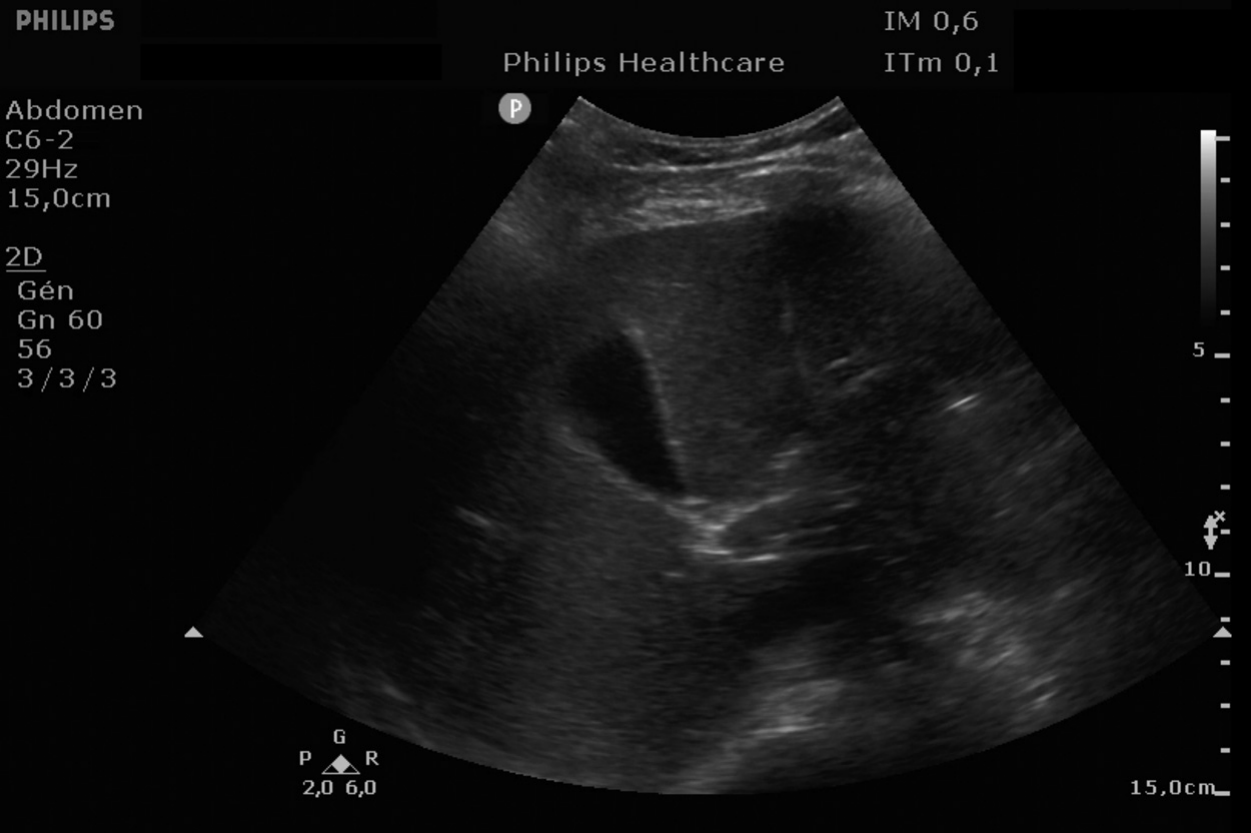
Upper abdominal longitudinal scan of the gallbladder

**Fig. 2:**
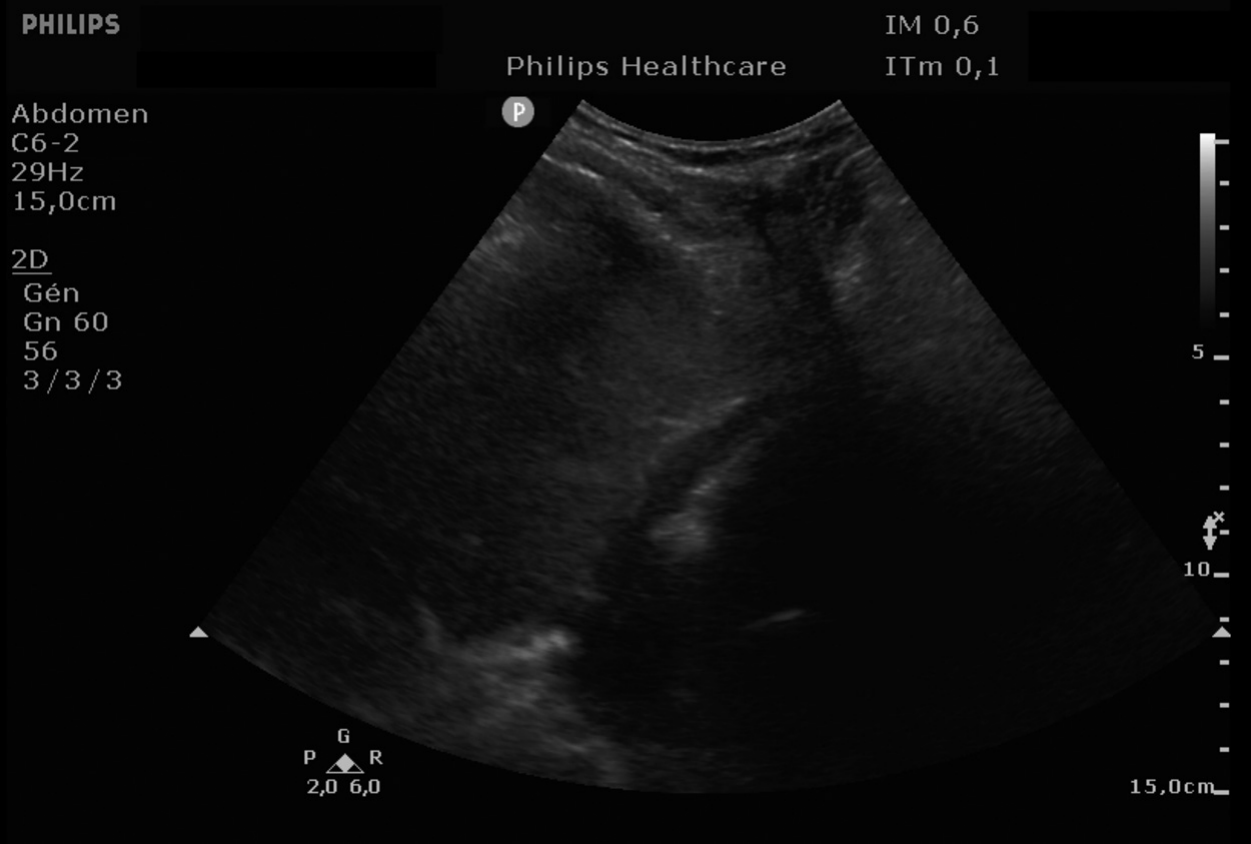
Pericardial fluid

**Fig. 3:**
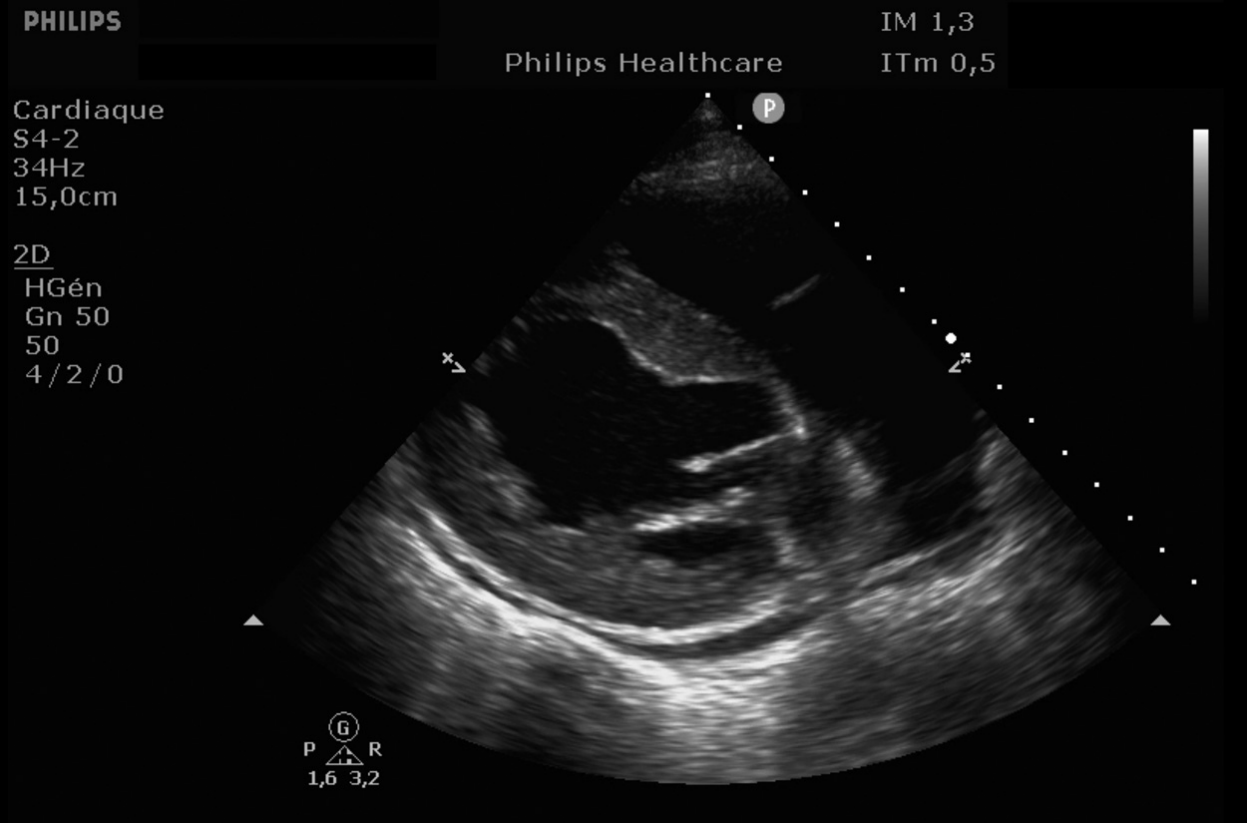
Parasternal long axis of the heart

**Fig. 4:**
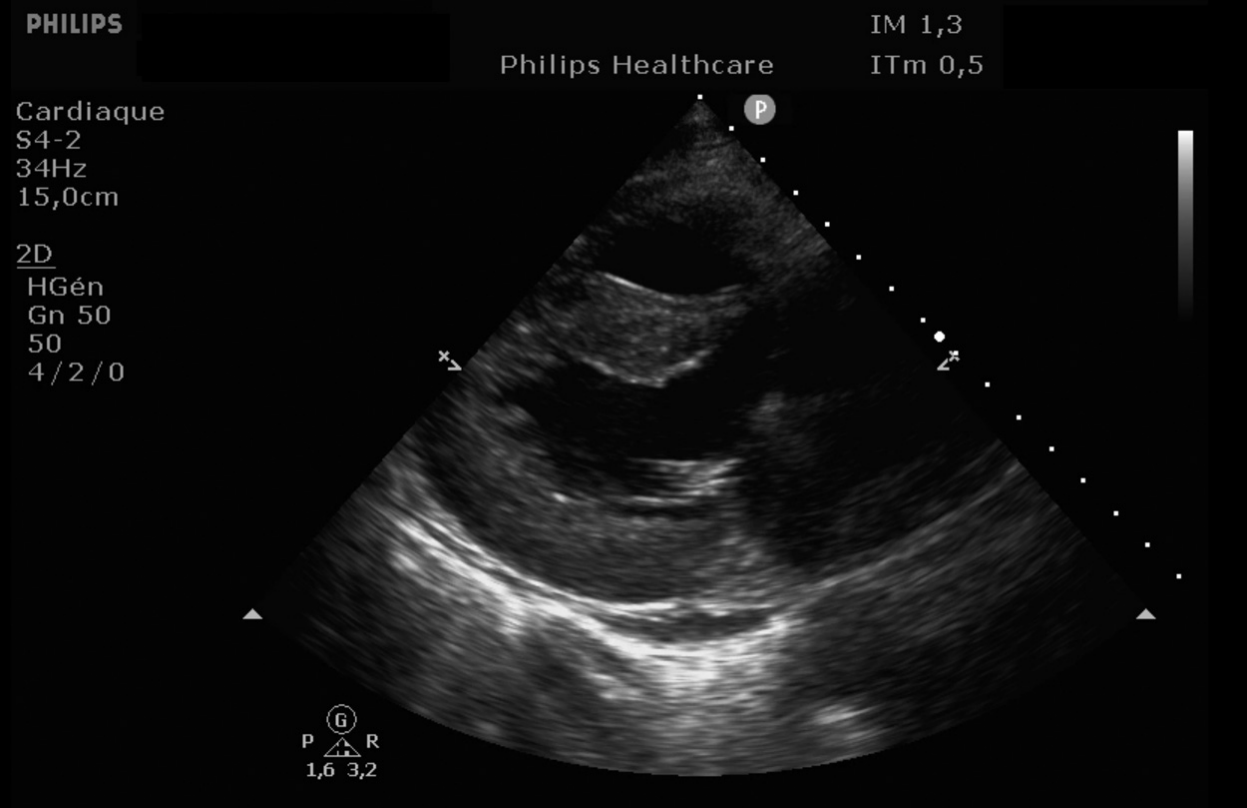
Parasternal long axis of the heart

**Fig. 5:**
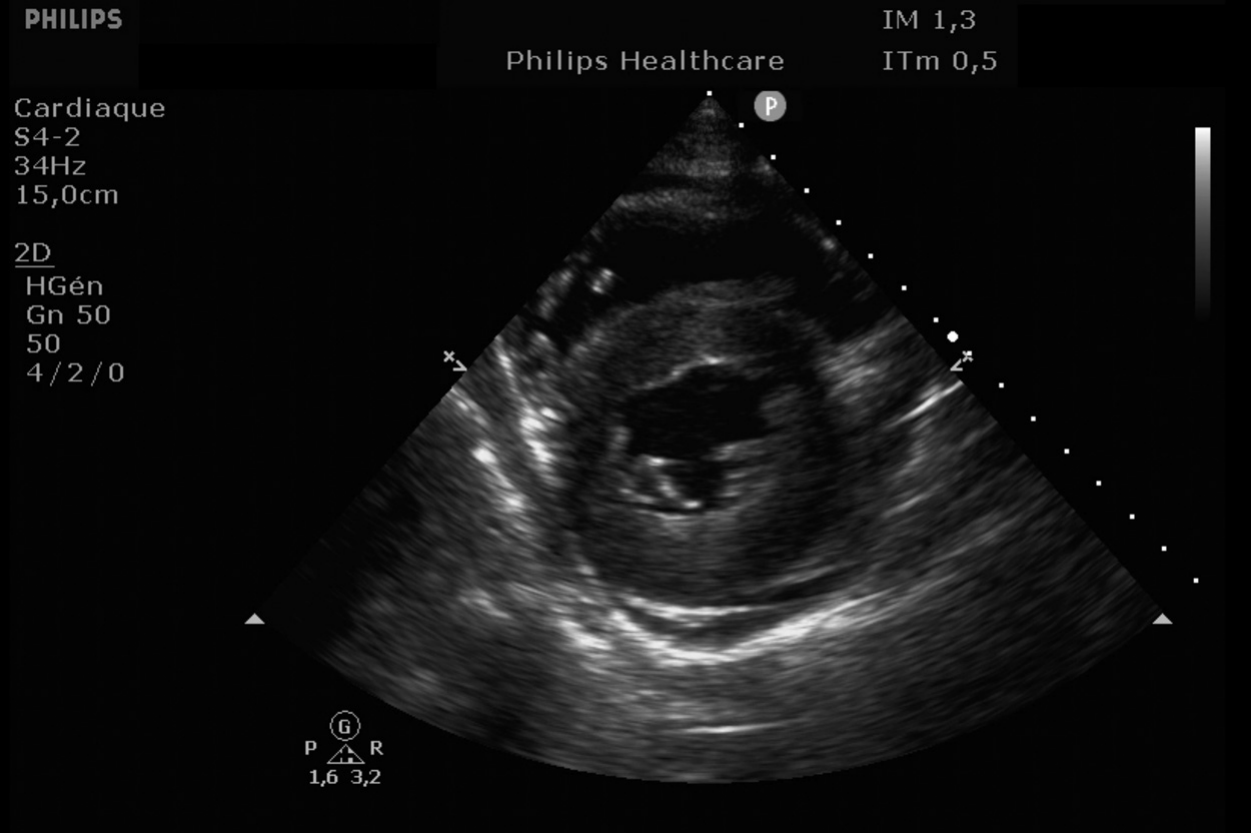
Parasternal short axis window

**Fig. 6:**
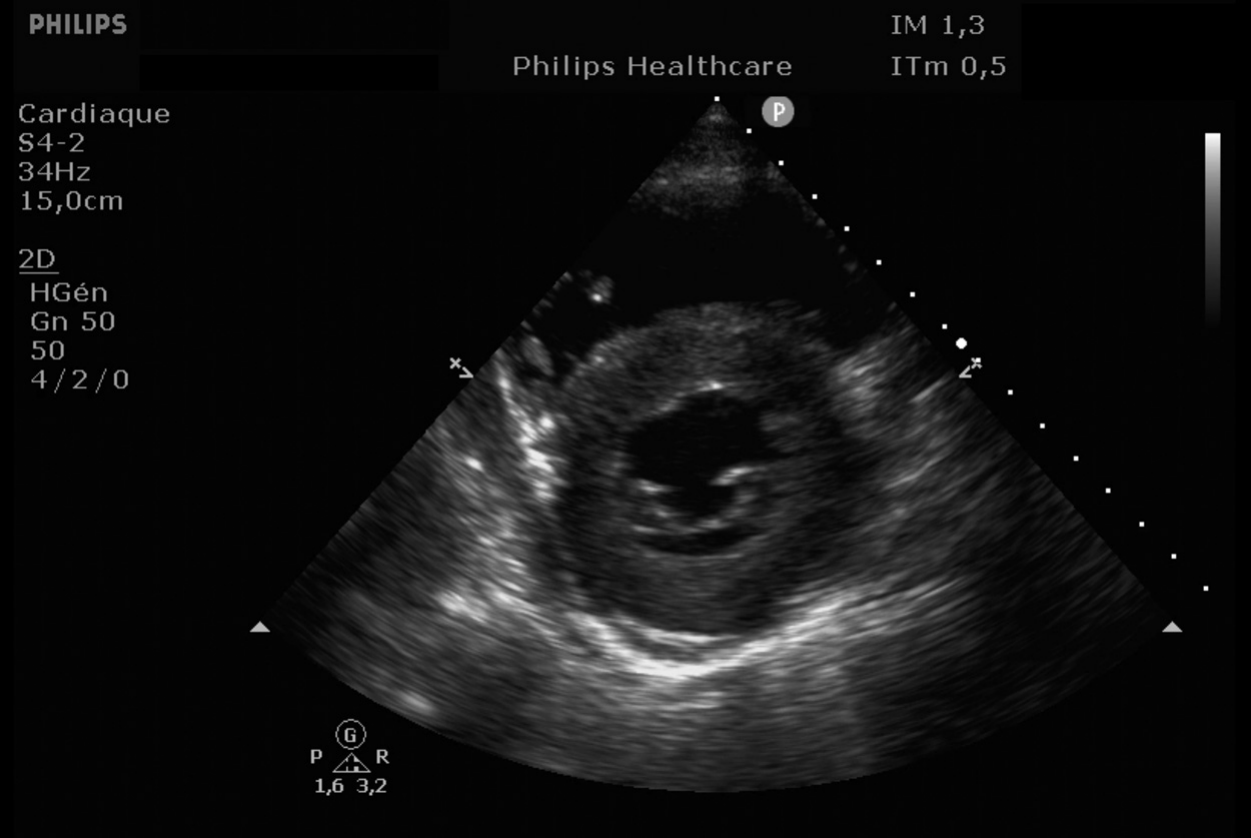
Parasternal short axis window

**Fig. 7:**
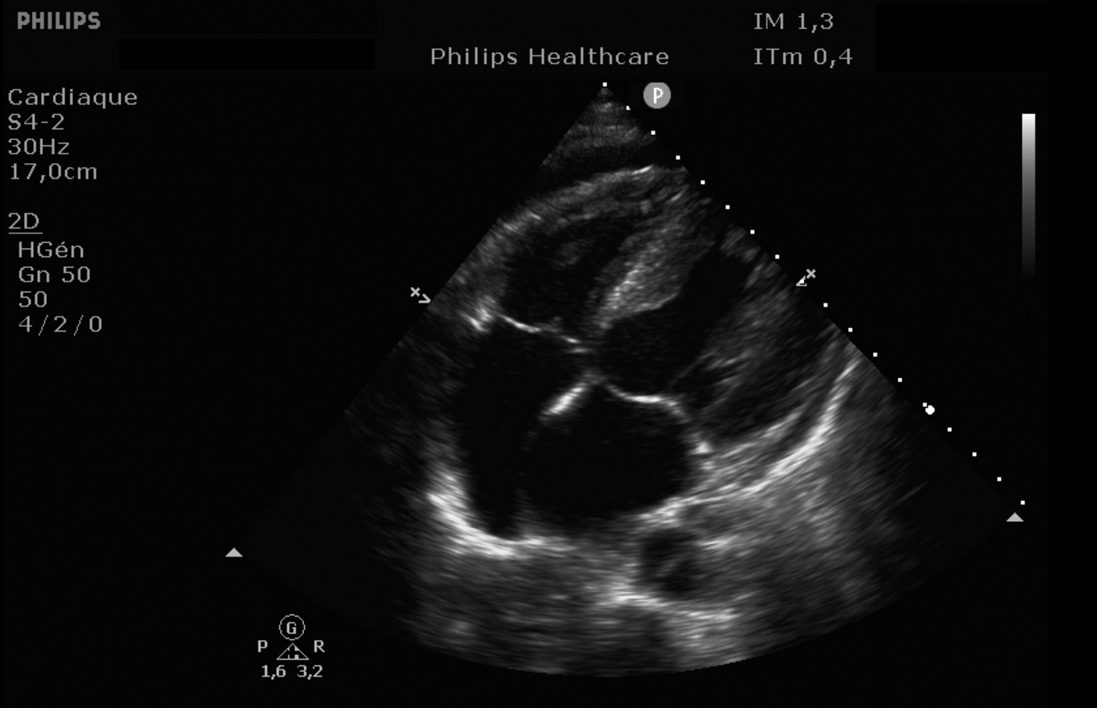
Apical view window

**Fig. 8:**
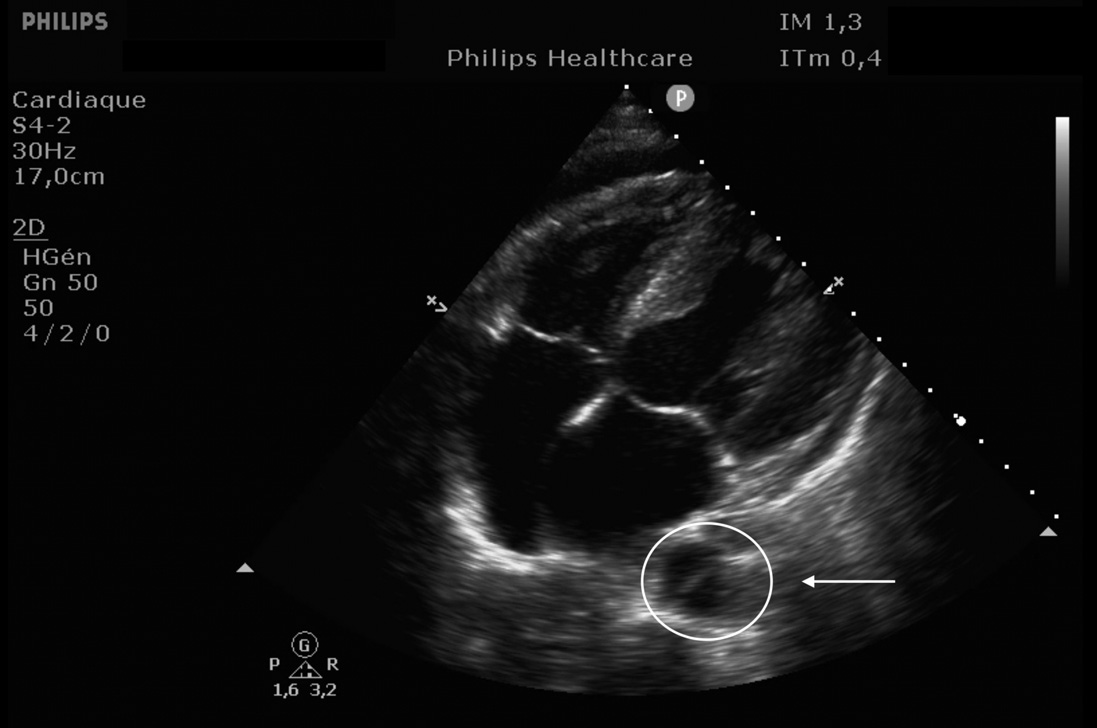
Apical View, Flap seen on the descending Aorta

**Fig. 9:**
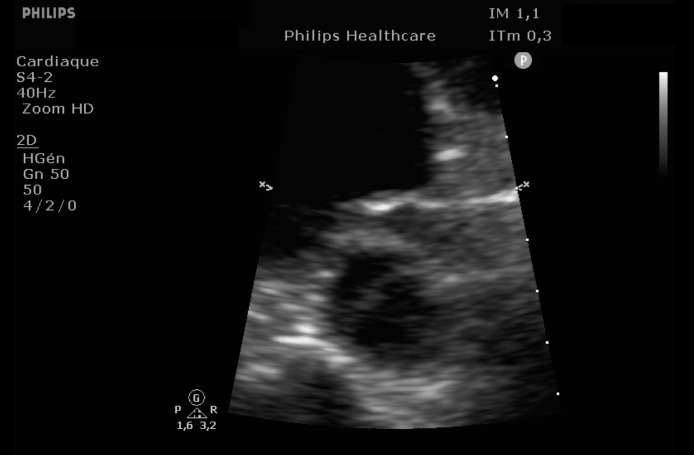
Apical View, Flap seen on the descending Aorta

**Fig. 10:**
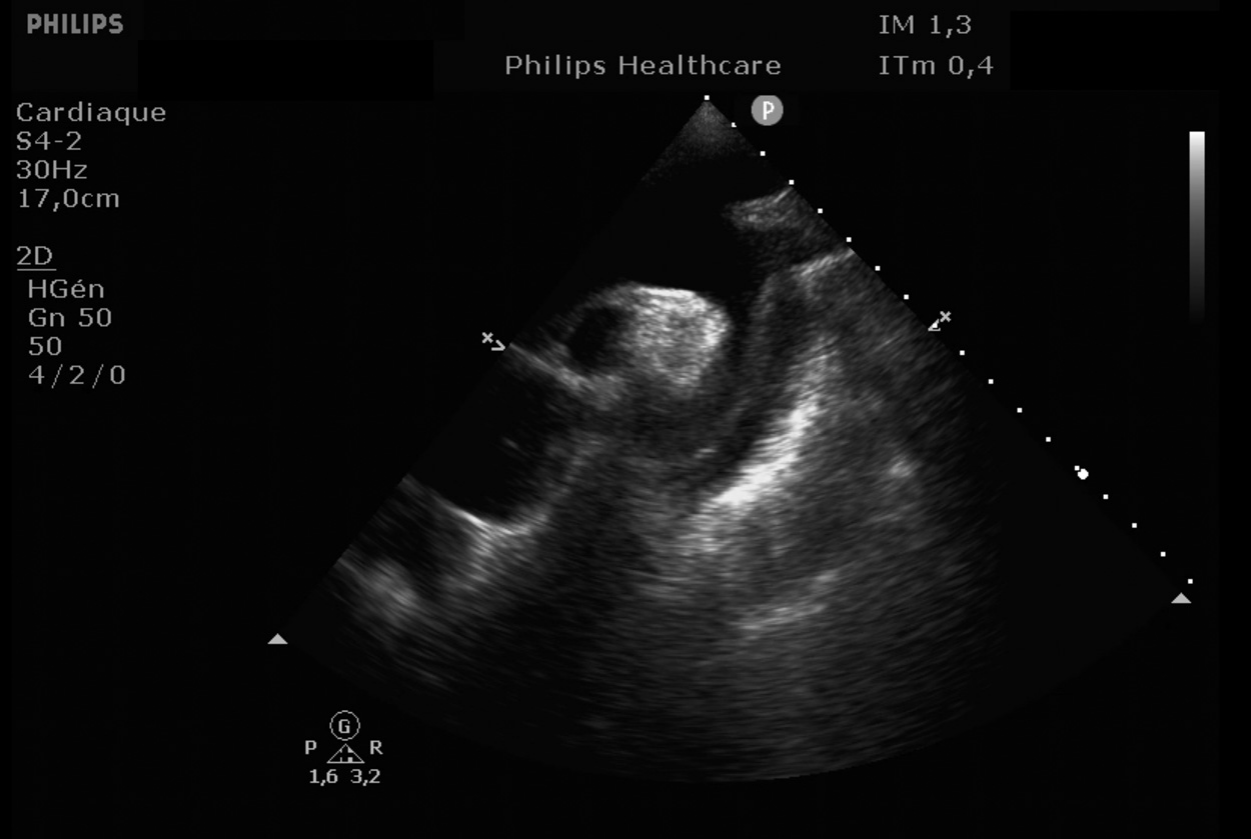
Suprasternal View, Aortic Flap

**Fig. 11:**
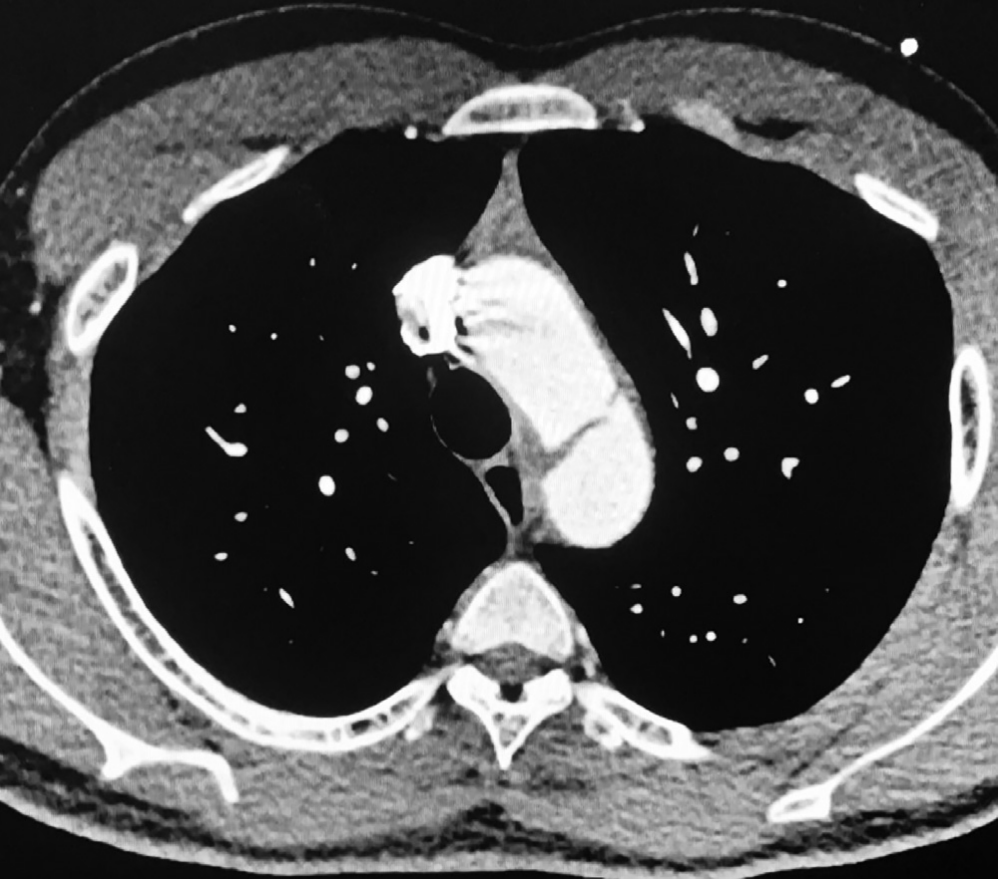
Aortic Dissection seen on CT Scan.

EKG showed sinus rhythm with signs of left ventricular hypertrophy, consistent with his chronic hypertension. Complementary laboratory exams were ordered and during the waiting time, point of care ultrasound was operated by the emergency physician to rule out gallbladder related problems. Ultrasound showed a normal gallbladder without gallstones, no wall thickening and no perihepatic or pericholecystic fluids (Fig. 1). With these normal findings, we proceeded to search for pericarditis, although the history and pain were atypical. A pericardial fluid was observed which led to a full cardiac ultrasound (Fig. 2). The parasternal long axis window (Figs. 3 and 4), parasternal short axis window (Figs. 5 and 6) and the apical view window (Fig. 7) showed left ventricle hypertrophy and the pericardial fluid. It was the apical view window that caught our attention (Fig. 8). The presence of a flap on the descending aorta was clearly seen (Fig. 9), it was confirmed on the suprasternal view (Fig. 10) allowing us to diagnose an aortic dissection (Fig. 11).

Vascular surgery team was notified, the patient was rapidly started on intravenous antihypertensive and rushed into radiology for CT Angiography for classification. CT showed Stanford B aortic dissection and a pericardial effusion of 2 mm of thickness. The patient was opted for a medical treatment and was admitted to the surgical unit for surveillance and continue of care.

The abdominal pain is a common presentation in the Emergency departments [2]. Even the most experience physician can be easily fool by a tunnel vision when it comes to abdominal pain in young adults. This case taught us how important it is to always have broad differential diagnosis even with the young population. In this situation, in a very busy emergency department, an aortic dissection would have been easily missed. The patient would have been discharged home with a diagnosis of gastritis as investigations were normal. This case highlights the importance of POCUS in the Emergency Department, and how it can easily change the course and management of patients when handled by well-trained physicians. If the physicians did not run his ultrasound on the gallbladder and fortunately recognized a pericardial effusion, a young patient with an aortic dissection would have been sent home, which would have a led to a terrible outcome.

## Discussion

Point of care Ultrasound is being more and more incorporated in medical schools’ curriculums and residency programs that manage acutely ill patients. The use of ultrasound in the emergency departments is increasing dramatically which can be, when used in the proper way, very helpful in diagnosing and eliminating probable differential diagnosis.

The most important thing to remember is that ultrasound is operator dependent, and it is crucial that every physician knows his limits when using the probes. Teaching point of care ultrasound for emergency medicine residents and physicians is essential, and can be, when used in an appropriate approach, an essential tool to diagnosis life threating events.

## Conflict of Interest

The authors declare no conflict of interest. The patient was contacted, and a written consent was taken to write this case report and to share the figures.
